# Central Nervous System Involvement of T-cell Prolymphocytic Leukemia Diagnosed with Stereotactic Brain Biopsy: Case Report

**DOI:** 10.4274/Tjh.2012.0028

**Published:** 2014-03-05

**Authors:** Selçuk Göçmen, Murat Kutlay, Alev Erikçi, Cem Atabey, Özkan Sayan, Aptullah Haholu

**Affiliations:** 1 Gülhane Military Medical Academy, Haydarpaşa Training Hospital, Department of Neurosurgery, İstanbul, Turkey; 2 Gülhane Military Medical Academy, Department of Neurosurgery, Ankara, Turkey; 3 Gülhane Military Medical Academy, Haydarpaşa Training Hospital, Department of Hematology, İstanbul, Turkey; 4 Gülhane Military Medical Academy, Haydarpaşa Training Hospital, Department of Pathology, İstanbul, Turkey

**Keywords:** T-cell prolymphocytic leukemia, Cerebral involvement, Central nervous system, Stereotactic biopsy

## Abstract

Prolymphocytic leukemia (PLL) is a generalized malignancy of the lymphoid tissue characterized by the accumulation of monoclonal lymphocytes, usually of B cell type. Involvement of the central nervous system (CNS) is an extremely rare complication of T-cell prolymphocytic leukemia (T-PLL). We describe a case of T-PLL presenting with symptomatic infiltration of the brain that was histopathologically proven by stereotactic brain biopsy. We emphasize the importance of rapid diagnosis and immediate treatment for patients presenting with CNS involvement and a history of leukemia or lymphoma.

## Abstract (tr)

Prolenfositik lösemi (PLL), genellikle B hücre tipi monoklonal lenfositlerin birikimi ile karakterize lenfoid dokunun genel malign bir hastalığıdır. Santral sinir sistemi (SSS) tutulumu T-hücreli Prolenfositik löseminin (T-PLL) çok nadir görülen bir komplikasyonudur. Biz, beyinin semptomatik infiltrasyonu ile ortaya çıkan, stereotaktik beyin biopsisi ile tanı koyulmuş T-PLL’li bir olgu sunuyoruz. Geçmişte lösemi veya lenfoma nedeni ile tedavi edilmiş ve SSS tutulumu ile başvuran hastalar için, hızlı tanı ve acil tedavinin önemini açıkladık.

## INTRODUCTION

Symptomatic central nervous system involvement (CNS) is a rare complication in T-cell prolymphocytic leukemia (T-PLL), although it is common in acute leukemia and non-Hodgkin’s lymphoma [1,2,3,4,5]. We report a case of T-PLL with symptomatic infiltration of the brain that was histopathologically proven with stereotactic brain biopsy.

## CASE REPORT

A 56-year-old man was admitted due to recent onset of severe headache. He was also noted to have multiple lymphadenopathy and hepatosplenomegaly. He had a history of T-PLL diagnosed 2 years ago. Bone marrow aspiration was a dry tap. Imprint was hypercellular and consisted of medium-sized prolymphocytes with single nuclei and basophilic cytoplasm with occasional blebs or projections. 

Laboratory data revealed leukocytosis (53x109/mm3) with normal values for hemoglobin (13.8 g/dL) and platelets (263x106/mm3). Differential blood count revealed 73% lymphocytes, 25% neutrophils, and 2% monocytes. In the flow cytometric examination of bone marrow, 80% of the lymphocytes were T-cells with co-expression of CD5, CD3, and CD52, as well as weak expression of CD7. No CD4, CD8, or other B cell markers were detected. β2-Microglobulin was elevated up to 2519 mg/L (normal:1310 mg/L). Direct Coombs test was negative and serum immunoglobulins were within normal limits. T lymphocytes were considered as leukemic infiltration. Lymphocytes demonstrated normal morphology. Surface marker analysis showed typical features of T-cell chronic lymphocytic leukemia (CLL) (83% of the cells CD3/CD5-positive). Biochemical profile was within normal limits. Informed consent was obtained.

**Figure 1:**
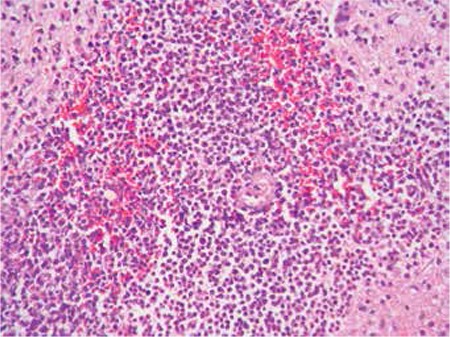
Hypercellular bone marrow infiltrated by the leukemia.

**Figure 2:**
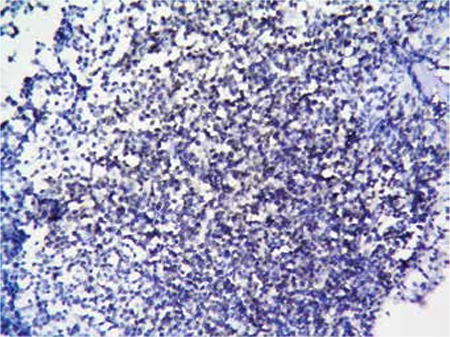
Immunohistochemistry staining CD3 expression of the leukemic cells.

**Figure 3:**
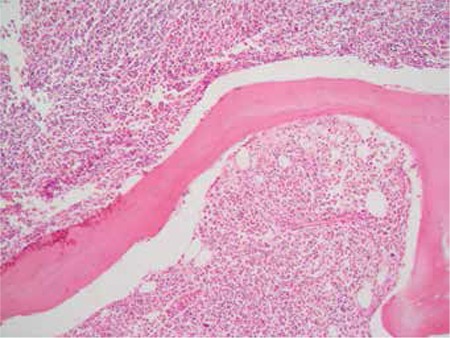
MPO expression in entrapped myeloid cells, while leukemic cells are negative.

Bone marrow biopsy revealed hypercellular bone marrow that was totally infiltrated by immature-appearing lymphocytes with prominent nucleoli (Figure 1). Immunohistochemical analysis demonstrated CD3 expression of the infiltrating cells (Figure 2). MPO expression was scarce in the myeloid cells entrapped in the leukemic infiltrate (Figure 3). 

The patient was given 3 courses of systemic chemotherapy consisting of fludarabine at 30 mg/m2 daily for 3 days intravenously and cyclophosphamide at 250 mg/m2 daily for 3 days on a 28-day cycle. He achieved hematological remission with no evidence of splenomegaly and had normal complete blood count values. In the interval between the third and fourth chemotherapy, the patient, who was previously asymptomatic, was admitted to the emergency unit with confusion, dysarthria, urinary incontinence, and generalized muscle weakness. His neurological examination was otherwise unremarkable. 

**Figure 4:**
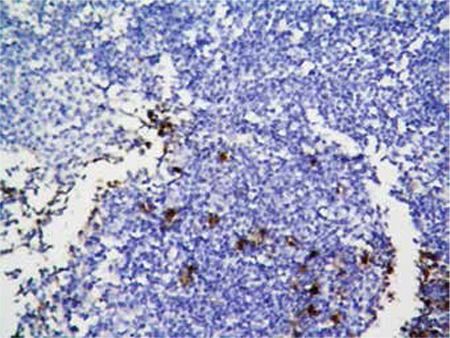
T1-weighted image (A) and T2-weighted magnetic image (B) show a focal lesion in the left frontal lobe.

**Figure 5:**
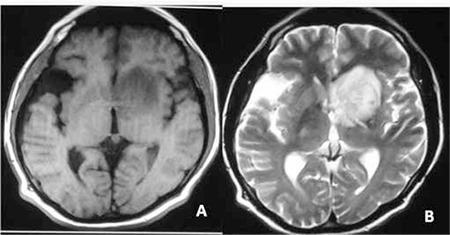
Prominently perivascular leukemic infiltration in the brain.

Emergency cranial computerized tomography (CT) was done, showing an infiltrating mass lesion and a right temporal arachnoid cyst. Magnetic resonance imaging of the brain revealed a focal lesion in the left frontal lobe with surrounding edema (Figure 4). Finally, the diagnostic work-up was completed with cervical-thoracic-abdominal CT that did not reveal any changes with respect to the patient’s previous condition. A stereotactic brain biopsy was performed. Brain tissue was also infiltrated by leukemia, which was especially prominent in the perivascular areas (Figure 5). Unexpectedly the immunohistochemistry revealed marked expression of T-cell markers (CD3, CD5, CD7). At that time, treatment with alemtuzumab was planned; however, the patient died before treatment could be started.

## DISCUSSION

T-PLL is rare, representing approximately 2% of cases of mature lymphocytic leukemias in adults over the age of 30, with a median age of 65 at presentation [6]. T-PLL is an aggressive T-cell leukemia characterized by the proliferation of small to medium-sized prolymphocytes with a mature post-thymic T-cell phenotype involving the peripheral blood, bone marrow, lymph nodes, liver, spleen, and skin [6]. 

Most patients present with hepatosplenomegaly and generalized lymphadenopathy. Skin infiltration is seen in 20% of patients, with occasional serous pleural effusions [6]. Anemia and thrombocytopenia are common and the lymphocyte count is usually >100x10^9^/L; it is >200x10^9^/L in half of the patients [6]. Serum immunoglobulins are normal [6]. The course of the disease is aggressive, with a median survival of usually less than 1 year [2]. The disease is often refractory to conventional chemotherapy (eg., alkylating agents or CHOP regimens), and it is considered incurable [2]. 

Direct symptomatic invasion of the CNS by CLL is extremely rare. To date, less than 30 cases have been reported in the literature, with various initial clinical manifestations, including headaches, confusional state, cranial nerve palsies, optic neuropathy, cerebellar dysfunction, or motor deficits, most often associated with leukemic meningitis [7]. In contrast, autopsy series have reported brain or spinal cord tumoral CLL involvement in 17% to 71% of cases, but with few clinical correlations [7]. Most of the CNS involvements were asymptomatic [7]. Garderet et al. reported that all cases with CNS involvement of PLL are of B cell origin; they have not been found to be associated with T-PLL. T-PLL of CNS is treated with non-myeloablative allogeneic stem cell transplantation [2]. Non-invasive diagnostic imaging techniques are usually inadequate for diagnosis. 

In spite of the most recent advances in diagnostic imaging, precise histopathological diagnosis is still critical for optimum treatment of these intracranial lesions. Many non-neoplastic lesions of the CNS may be misinterpreted to be tumors due to their clinical and radiological presentation, with patients subjected to unnecessary surgical treatment, until histopathological diagnosis is established [8]. Most of these patients are best managed with medical therapy alone. Likewise, certain neoplastic processes, such as lymphoma or germinoma, respond very well to chemotherapy. Surgery is also of minimal benefit in most brain metastasis or advanced primary tumors (glioblastoma multiforme). These patients can be better served with radiation and/or chemotherapy. 

Stereotactic needle biopsy is a safe approach to establish the histopathological diagnosis for most intracranial lesions, especially for deep-seated, adjacent to eloquent areas, brain stem, or multiple small lesions [9,10,11]. Patients’ general medical condition and co-morbidities should also play a role in preferring stereotactic biopsy over open surgery [11]. 

Frame-based or frameless image-guided stereotactic brain biopsies were reported to have high diagnostic yields, ranging between 85% and 98% [10]. Complication rates range from 2% to 6.5%, and most complications did not result in clinically significant consequences [10]. 

Dammers et al. reported that frame-based and frameless image-guided stereotactic brain biopsy techniques are not different (i. e. equivalent) [12]. Reported overall morbidity rate is 6.9% and mortality rate 1.3% for frame-based stereotactic biopsy [13]. Intracranial hemorrhage rates from 0% to 9% have been reported in the literature [13]. Frame-based or frameless stereotactic brain biopsy of an intracranial lesion is a safe surgical technique with high diagnostic yield and low morbidity and mortality [10,11,12,13]. It should be the procedure of choice in establishing histopathological diagnosis and planning the extent of surgical treatment. 

CNS leukemia is a rapidly progressive disease with risk of dismal outcome. Treatment of CNS leukemia is by steroids, intrathecal or systemic chemotherapy, cranial irradiation, or a combination of these [14,15,16,17,18]. Nucleoside analogs and immunotargeting therapies are the most widely used types of treatment, but the results are frequently temporary. There is no standard treatment for CNS involvement of T-cell lymphoid malignancies, and generally this condition is considered incurable. Our patient had a rare presentation of relapse appearing in the CNS. Immediate and accurate histopathological diagnosis is crucial for treatment in patients presenting with CNS involvement and a history of leukemia or lymphoma. A stereotactic biopsy should also be considered for medically unstable patients and patients with inoperable CNS malignancies. 

## CONFLICT OF INTEREST STATEMENT

The authors of this paper have no conflicts of interest, including specific financial interests, relationships, and/ or affiliations relevant to the subject matter or materials included.
